# Impact of Stress Levels on Eating Behaviors among College Students

**DOI:** 10.3390/nu12051241

**Published:** 2020-04-27

**Authors:** Jinkyung Choi

**Affiliations:** Foodservice Management, Woosong University, Daejeon 34606, Korea; choi3728@wsu.ac.kr

**Keywords:** stress, eating behaviors, PSS-10, college students

## Abstract

Weight gain is a common phenomenon among college students, especially those in their first year of university. Transitioning from high school to the college environment might increase perceived stress levels, thus affecting dietary behaviors and metabolism to promote overweight and obesity. The purpose of this study was to investigate the physical activities and dietary behaviors of college students in the context of their perceived stress levels. In addition, the demographic characteristics of the students were compared to ascertain their impact on dietary behaviors. Self-reported questionnaires were distributed to college students on campus in Korea. Perceived stress was measured by the 10-item Perceived Stress Scale (PSS-10), the scores for which were evaluated by independent *t*-tests to compare the dietary behaviors of the high- and low-perceived stress groups. Exploratory factor analysis was performed and Cronbach’s alphas were computed to assess the validity and internal consistency of the PSS-10 measurement items. Differences in the physical activities and dietary behaviors of the college students based on demographics such as sex, academic year, and residence type were found. Several dietary behaviors were significantly different between students with low and high perceived stress levels. Students with high perceived stress levels exhibited increased unhealthy dietary behaviors such as ready-prepared meal consumption (*p* < 0.001). These results suggest that stress management should be offered to college students. In addition, programs should be provided to help first-year students adjust to the college environment in order to promote healthy dietary behaviors.

## 1. Introduction

Being physically and psychologically healthy has been shown to improve quality of life. Despite the benefits of being healthy, the rate of obesity has increased two- to three-fold in the last 20 years [1]. Notably, data published in 2009 by the American College Health Association’s National College Health Assessment indicated that 36.7% of college students were overweight or obese [2]. When investigating the relationship between sleep and obesity among U.S. and South Korean college students, Sa [3] observed higher overweight/obesity rates (59.4%) in Korean males than Black (51.5%) and White males (46.8%) and in Black women (53.8%) than in White (38.8%) and Korean women (24.8%) based on the standard body mass index (BMI) cut points for U.S. students and Asian BMI cut points for Korean students.

Dietary habits are influenced by various genetic, sociodemographic, and environmental factors. Food preferences developed and established in childhood may change during the college years because of decreased parental influence [4,5,6,7,8]. An abundance of literature demonstrates that most students gain weight during the first year of college [9,10,11]. During the transition from high school to university, the failure of college students to adjust to the new environment and associated stressors can lead to negative dietary habits such as excessive alcohol consumption and stress-related under- or over-eating [4]. 

College students have lifestyles and dietary habits that differ from those of the general population, often relying on meals they can access quickly and easily [12]. Besides taste, convenience is the most important motivator for food choices [12,13]. Thus, fast food consumption is common among the dietary habits of college students [12,14,15,16]. Furthermore, most college students ignore the food group recommendations [17], frequently skip meals, have inappropriate snacks, and consume excessive amounts of alcohol [6]. Inconsistencies in food choices have been observed between the sexes. In some studies, female students tended to avoid fat, eat fruit [12], and were less likely to purchase high-energy foods [18], whereas male students were more likely to purchase alcoholic beverages [18]. Conversely, another study showed similar fruit and vegetable consumption among female and male students, but female students ate more fatty foods than males [19]. Perhaps these differences depend on the individual’s level of perceived stress because stress can affect eating behaviors [20]. For instance, stress can increase one’s consumption of snacks [21,22,23].

Stress is defined as a disturbance to one’s “physiological homeostasis or psychological well-being” [24]. A cross-sectional study of first-year university students studying at an Australian university found that more than half of the students were experiencing some degree of stress, with comparatively more women suffering than men, and that stress was associated with the selection of less healthy food [25]. There is also evidence that women under stress seek comfort from highly palatable foods or snack foods [26]. Another study showed that when under stress, people chose “unhealthy food” (e.g., high-calorie, high-fat snack food, sugary food, sweets [21,27], and carbohydrate-rich food [27]) rather than healthy food [21]. Nonetheless, the associations between eating behaviors, stress, and sex are inconclusive. In some individuals, stress increased food intake, while others displayed decreased food intake [26,28,29].

Recent research has recognized the importance of the analysis of dietary or food consumption patterns for assessing the overall diet [30,31]. Therefore, this study investigated the college students’ physical activities, dietary behaviors, and perceived level of stress in order to offer suggestions that support a healthier diet for college students, who were most lacking in sufficient nutrient intake and were vulnerable to stress. First, this study measured and compared the physical activities and dietary behaviors based on the respondents’ demographic characteristics. Second, the stress level of each participant and its association with physical activities and dietary behaviors were investigated.

## 2. Materials and Methods

### 2.1. Participants

Questionnaires were distributed for two months on a university campus in Korea. In 2019, when this study was carried out, there were 9725 students including 5170 males (53%) and 4555 females (47%), enrolled at the university. The study was approved by the institutional review board (1041549-191011-SB-80). Of the 420 questionnaires distributed to students enrolled in a 4-year college program, 396 questionnaires were collected. The students were informed about the study’s purpose and gave consent to participate before the survey proceeded. After screening, three questionnaires were excluded because of inappropriate responses, and the remaining 393 were retained for further analysis. 

### 2.2. Measurements

Nineteen questions regarding physical activity and dietary habits such as “I usually exercise regularly each week” and “I usually eat more than two pieces of fresh fruit per week” were adopted from previous studies [32,33]. Responses were rated on a 5-point Likert scale ranging from 0 (zero times per week) to 4 (at least seven times per week). The Perceived Stress Scale (PSS) is a valid and reliable instrument to measure stress [34,35,36]. Of the three PSS versions, which are comprised of 4-, 10-, and 18-item measurements, the PSS-10 has the most satisfactory psychometric properties [35,37]. The PSS-10 consists of six positively worded items and four negatively worded items. A two-factor PSS-10 model (perceived helplessness as a positive factor and perceived self-efficacy as a negative factor) has been identified [35]. The perceived stress level was evaluated by the PSS-10 [34,38]. Each item in the PSS-10 is rated on a 5-point Likert scale ranging from never (0 points) to very often (4 points). The last part of the questionnaire sought to obtain demographic information, establish whether the participant was on a diet, and collect weight and height data to calculate the BMI of each participant.

### 2.3. Statistical Analysis

Data were coded in Excel and then analyzed using the Statistical Package for the Social Sciences (SPSS) [39]. Descriptive analyses were conducted to determine the patterns of college students’ dietary behaviors and demographic information. Independent *t*-tests were run on dietary behaviors by sex (one-sided). Tukey’s test was undertaken on dietary behaviors according to each respondent’s academic year and residence type. Multiple regression was performed on BMI, with dietary behaviors as the independent variables. The construct validity of the PSS-10 was assessed by exploratory factor analysis using maximum likelihood estimation. Cronbach’s alphas (>0.60) were computed to measure the internal consistency of the PSS-10 factors. Independent *t*-tests were performed to investigate dietary behavior differences between the low- and high-stress groups. In addition, stress differences were measured for sex, academic year, and residence type. Alpha was set at 0.05 for all statistical tests.

## 3. Results

The demographic characteristics of the respondents are shown in Table 1. Males represented 45.2% of the respondents and 54.8% were female. Approximately 35% of the respondents were freshmen, and 31% were sophomores. Most of the respondents answered that they lived alone (71.2%). Only one-third of the respondents (24.2%) were on a diet. BMI was calculated based on self-reported height (cm) and weight (kg). BMIs (kg/cm^2^) < 18.5, 18.5–22.9, 23–24.9, and >25 were defined as low weight, normal weight, overweight, and obese, respectively [40]. About 50.3%, 18.3%, 13.6%, and 17.8% of the respondents were normal weight, overweight, low weight, and obese, respectively. The mean BMI was 20.854 (SD = 7.074), thereby falling in the normal weight range. 

Sex differences in BMI were significant (*t* = 7.722, *p* < 0.001). Males (M = 23.683, SD = 4.046) had a higher BMI than females (M = 18.462, SD = 8.134), who, in general, fell in the low-weight range. Students who were on a diet had a significantly (*t* = 3.369, *p* = 0.001) higher BMI (M = 22.894, SD = 6.501) than those not on a diet (M = 20.193, SD = 7.167). Significant differences were found among the academic years of the students (F = 4.186, *p* < 0.01). Freshmen (M = 22.470, SD = 5.094) had a higher BMI than sophomores (M = 19.488, SD = 6.784).

Table 2 displays the physical activities, alcoholic beverage consumption, and dietary behaviors of the respondents. The majority of the respondents exercised 1–2 times a week (43.8%) for more than 30 min, whereas 42% did no exercise. A total of 46.1% of the respondents consumed alcoholic beverages 1–2 times a week, and more than half of the respondents were not taking dietary supplements (54.5%). Nearly 47% of the respondents answered that they ate when stressed. The respondents reported that they ate the following foods 1–2 times per week: over 40% ate more than two pieces of fresh fruits, nearly 44% ate fish and seafood, and over 43% ate dairy products such as milk, yogurt, and cheese (43.3%). Meats (39.7%) and bread or noodles made with wheat (43%) were consumed as a meal 3–4 times a week. Most of the respondents had snacks such as cakes, candies, and soft drinks (40.4%), fruit, milk, and nuts (41.1%), and ready-prepared meals (41.4%) 1–2 times a week. Fast food consumption 1–2 times a week had the highest frequency (51.7%), and 33.8% of the respondents ate out 3–4 times a week. Some 33.4% of the respondents skipped breakfast seven times a week, but most did not skip lunch (49%) or dinner (55.2%). The frequency of overeating 1–2 times a week was 49.4%.

Dietary habits were compared between sexes (Table 3). More males exercised regularly (M = 2.21, SD = 1.019, *t* = 5.823, *p* < 0.001) and for at least 30 min (M = 2.14, SD = 1.040, *t* = 5.701, *p* < 0.001) than females (M = 1.67, SD = 0.766; M = 1.60, SD = 0.780). However, more females ate to release stress (M = 2.30, SD = 0.879, *t* = −2.263, *p* < 0.05) and consumed more than two pieces of fresh fruit (M = 2.16, SD = 1.022, *t* = −2.168) than males (M = 2.09, SD = 0.915; M = 1.94, SD = 0.984). Males consumed more meat (M = 3.35, SD = 0.998, *t* = 6.173, *p* < 0.001) and fish/seafood (M = 1.94, SD = 0.828, *t* = 3.311, *p* < 0.01) than females (M = 2.77, SD = 0.863; M = 1.67, SD = 0.777). Unhealthy dietary behaviors were more prevalent in females, as they more often had snacks such as cakes, candies, and soft drinks (M = 2.73, SD = 0.984, *t* = −2.368, *p* < 0.05), and skipped dinner (M = 1.73, SD = 0.917, *t* = −2.068, *p* < 0.05) than males (M = 2.49, SD = 1.005; M = 1.55, SD = 0.820).

There were significant differences in the students’ physical activities and dietary behaviors based on academic year. Exercising for more than 30 min was less prevalent among freshmen (M = 1.82, SD = 0.791, *F* = 4.245, *p* < 0.01) than seniors (M = 2.27, SD = 0.919). Freshmen also consumed fewer dietary supplements (M = 1.88, SD = 1.230, *F* = 4.343, *p* < 0.01) than seniors (M = 2.81, SD = 1.600). Moreover, freshmen (M = 1.60, SD = 0.637, *F* = 4.074, *p* < 0.01) consumed fewer alcoholic beverages than sophomores (M = 1.83, SD = 0.874) and juniors (M = 1.88, SD = 0.813). Sophomores (M = 2.21, SD = 1.044, *F* = 3.999, *p* < 0.01) had a higher intake of snacks such as fruit, milk, and nuts than juniors (M = 1.85, SD = 0.954), who in turn, had the lowest consumption of such snacks.

Dietary behaviors differed significantly based on residence type. Students who lived with their parents were more likely to consume more than two pieces of fresh fruit per week (M = 2.52, SD = 1.114) than those who lived on their own (M = 1.87, SD = 0.891) and showed higher fish intake (M = 2.08, SD = 0.851 vs. M = 1.67, SD = 0.755). Students who lived on their own (M = 1.96, SD = 0.907) had a lower intake of snacks such as fruit, milk, and nuts when compared with those who lived with their parents (M = 2.33, SD = 1.106) and those who lived in the dormitory (M = 2.94, SD = 1.144). The same patterns were noticed regarding skipping breakfast and lunch. Respondents who lived on their own skipped breakfast (M = 3.60, SD = 1.392) and lunch (M = 1.95, SD = 1.054) more often relative to those who lived with their parents (M = 2.77, SD = 1.492; M = 1.57, SD = 0.865).

Multiple regression was conducted on BMI as the dependent variable and dietary behaviors as the independent variables. The model (F = 2.622, *p* < 0.001, R^2^ = 0.126, adjusted R^2^ = 0.078) showed that the intake of meat (B = 1.831, SE = 0.444, *t* = 4,124, *p* < 0.001) and fast food (B = 1.583, SE = 0.560, *t* = 2.826, *p* < 0.01) had a positive effect on BMI, whereas the intake of snacks such as cakes, candies, and soft drinks (B = −1.371, SE = 0.433, *t* = −3.167, *p* < 0.01) negatively affected BMI.

The PSS-10 items are shown in Table 4. Exploratory factor analysis was conducted and Cronbach’s alphas were computed to assess the validity and reliability of the PSS-10 measurements. Two factors were extracted, of which one was positively coded (six items) and one was negatively coded (four items). All PSS-10 items were added and each total score was then divided by its mean value (M = 18.425, SD = 5.279). Results below the mean value were coded as “1” (“low stress”) and results above the mean value were coded as “2” (“high stress”). Independent *t*-tests were conducted on the dietary behaviors of the low- and high-stress groups (Table 5), which revealed that the high-stress group (M = 1.81, SD = 0.854) performed less regular exercise (M = 2.02, SD = 0.981, *t* = 2.137, *p* < 0.05). In addition, the high-stress group (M = 2,43, SD = 0.933) had a higher frequency of eating to release stress than the low-stress group (M = 1.94, SD = 0.781, *t* = −5.556, *p* < 0.001) as well as a higher frequency of eating fast food, ready-prepared meals, and snacks (e.g., cakes, candies, and soft drinks); skipping meals (breakfast, lunch, and dinner); and overeating. However, meat consumption was higher in the low-stress group (M = 3.12, SD = 0.982) than the high-stress group (M = 2.93, SD = 0.956). A significant difference in stress was found based on sex (*t* = −4.049, *p* < 0.001), with a higher perceived stress level in females (M = 19.379, SD = 4.876) than males (M = 17.216, SD = 5.486) (not shown in table). The stress level did not differ with the respondents’ academic year, residence type, or dieting.

## 4. Discussion

This study found several characteristics of college students’ dietary behaviors including a strong reliance on meat consumption and eating out 3–4 times a week. The frequency meat intake was higher than that of fish/seafood and fresh fruit. The majority of the respondents did not exercise regularly or take dietary supplements. One-third of the respondents skipped breakfast almost every day, but few skipped lunch and dinner. Skipping meals has been associated with unhealthy dietary behaviors [41]. 

The observed dietary behaviors differed based on the students’ sex, residence type, and academic year. More male students exercised regularly and for more than 30 min when compared with female students. Moreover, meat and fish/seafood consumption was more prevalent among males than females. Female students tended to eat to release stress, which supports the previous findings that females are more likely to be emotional eaters than males [21,42]. Interestingly, some studies have suggested that females eat more fresh fruit [12] and consume more snacks such as cakes, candies, and soft drinks than males [22]. Overall, the male students in this study tended to exhibit healthier dietary behaviors than the females, in contrast to a previous study [25]. 

Differences in the students’ dietary behaviors based on academic year suggest the potential of academic institutional support and educational interventions. Some surveys have shown that first-year students display a low frequency of regular exercise, and it has been suggested that they fail to adjust to the new environment [4,20]. Hence, college administrations should provide assistance that supports first-year college students in their transition to the institution as well as implement recommended educational interventions regarding nutritional information, disseminated via leaflets or posters, to reinforce healthy dietary behaviors.

The majority of the respondents answered that they lived alone, which was the same as the general college population [43]. However, great differences were observed in the dietary behaviors of the students based on residence type [44]. Students who lived with their parents displayed much healthier dietary behaviors than those who lived on their own. For most students, this is their first time away from home, and so might lack the time, organization, or cooking skills to prepare meals [15]. Providing easy-cooking leaflets would encourage college students to cook for themselves and choose better food options by decreasing their reliance on eating out, fast food, and ready-prepared meals.

In this study, the average stress score was 18.43, which was higher than that reported by a group of infertile women (17.48) [35] and the general population (13.02) [36]. Adjusting to the college environment can be highly stressful, and stress greatly influences dietary behaviors. The high-stress group exhibited fewer healthy dietary behaviors as they tended to eat more sugar-based snacks, carbohydrate-rich food [21,27], fast food, and ready-prepared meals such as comfort food when compared with the low-stress group [26]. The results suggest that college students showed two opposing behaviors under stress including the more frequent intake of certain types of food (overeating) and less frequent eating such as skipping meals (undereating). 

Two factors of stress are perceived helplessness and self-efficacy. These factors may increase eating in general as well as irregular eating, which can explain stress-induced overeating and undereating behaviors [45]. In addition, skipping meals is associated with stress and depressed mood [46], which may mean that skipping meals can be both the cause and result of stress. Regular breakfast consumption may increase physical activity [47], and so encouraging college students to eat breakfast is necessary for their health. Females have been shown to have a higher perceived stress level than males [25] and be more likely to diet. Moreover, distressed females are more likely to engage in binge eating [48] and be loss-of-control eaters [49]. Dietary restraint predicts overeating when under perceived stress [42] for both males and females [49]. Strict limitations on certain foods increase overeating in stressed individuals, so both female and male students should be informed about reasonable food consumption [48]. 

Dietary habits related to lifestyle are key elements of healthy living. Poorer mental and physical health-associated quality of life is related to body dissatisfaction, which is also associated with psychological distress [48,49]. For this reason, further studies should investigate the influence of internal and external lifestyle factors on dietary behaviors. Moreover, information on stress management should be provided to students, considering that high-stress students tend to eat when stressed. Emotional eating should be self-monitored and alternative stress-management strategies should be exercised for stress relief. University-affiliated clubs and societies that reflect the students’ interests may help in this regard. The availability of a variety of healthy food options on campus including in cafes and vending machines, along with nutritional information, could prevent strict food limitations. In times of stress, food that is usually prohibited or limited is consumed, so a balanced intake of food from all food groups should be followed in daily life.

The BMI of the respondents in this study was normal and greatly affected by the intake of meat and fast food. A previous report suggested that students eat fast food because of its low cost and convenience [12]. A lack of cooking ability may explain the high consumption of fast food and meat [15]. Regarding cooking, meat can be cooked more easily compared with other food ingredients. In addition, a Westernized lifestyle means that college students eat more meat than fish/seafood and fruit. Unexpectedly, the higher consumption of snacks including sugar-based snacks was associated with a low BMI, implying that students eat snacks as replacements for proper meals, resulting in a relatively lower overall calorie intake.

This study has some limitations to be improved for future research. As this study was conducted with students attending a single college campus in Korea, a geographical limitation exists. Most students in this study lived alone, whereas different results would be expected among students living with family. Stress levels associated with long commutes, a factor not examined in this study, may also affect outcomes. More college students should be included for further analysis because the students’ perceived stress levels may decrease with time spent at university as they adjust to the environment. This study used the frequency of intake of particular foods to determine dietary behaviors, whereas neither the amount of consumed food nor caloric information was considered. For further research, various food behaviors should be included to detect dietary behaviors more accurately. Moreover, the internal and external influences of dietary behaviors should be examined in the context of perceived stress levels. For example, body dissatisfaction, eating disordered behavior, and emotional eating should be assessed in future studies.

## 5. Conclusions

In this study, college students displayed a variety of dietary habits that differed between males and females. Moreover, differences were found in dietary habits based on the students’ academic year and residence type. It is important to investigate and understand the environments that affect college students’ dietary behaviors because eating patterns at this time in one’s lifespan can affect health and food behaviors throughout adulthood. Support from college administrations such as educational interventions is necessary to improve unhealthy food behaviors. Moreover, measured stress was relatively high for college students, implying they were under stress due to the new academic environment. Students with high stress showed less healthy dietary behaviors compared to students with low stress. Thus, college students’ stress should be managed properly to prevent unhealthy dietary behaviors related to stress. Stress management needs to start before college life, as stress should not hinder the college students’ ability to engage in healthy dietary behaviors.

## Figures and Tables

**Table 1:** Characteristics of respondents regarding demographics, dieting, and BMI (*n* = 393).

Characteristics		Frequency	Valid Percentage (%)
Sex	Male	176	45.2
	Female	213	54.8
	Missing	4	
Academic year	Freshman	136	35.0
	Sophomore	121	31.1
	Junior	106	27.2
	Senior	26	6.7
	Missing	4	
Place of residence	Board and lodging	4	1.0
	Alone	275	71.2
	School dormitory	17	4.4
	With parents	90	23.3
	Missing	7	
Are you on a diet?	Yes	93	24.2
	No	292	75.8
	Missing	8	
BMI	<18.5	52	13.6
	18.5–22.9	192	50.3
	23–24.9	70	18.3
	≥25	68	17.8
	Missing	11	

**Table 2:** Characteristics of respondents regarding physical activities, alcoholic beverage consumption, and dietary habits.

I Usually…per Week	≥7	5–6	3–4	1–2	0	Mean	SD
Exercise regularly	9 (2.3)	17 (4.3)	51 (13)	172 (43.8)	144 (36.6)	1.92	0.933
Exercise more than 30 min	10 (2.5)	15 (3.8)	44 (11.2)	159 (40.5)	165 (42)	1.84	0.944
Drink alcoholic beverages	4 (1)	7 (1.8)	46 (11.7)	181 (46.1)	155 (39.4)	1.79	0.795
Take dietary supplements	38(9.7)	30(7.6)	35 (8.9)	76 (19.3)	214 (54.5)	1.99	1.347
Eat when I am stressed	6 (1.5)	25 (6.4)	93 (23.7)	183 (46.8)	84 (21.5)	2.20	0.900
Eat more than two pieces of fresh fruit	10 (2.5)	29 (7.4)	64 (16.3)	160 (40.7)	130 (33.1)	2.06	1.009
Eat meat	32 (8.1)	82 (20.9)	156 (39.7)	110 (28.0)	13 (3.3)	3.03	0.974
Eat fish and seafood	2 (.5)	12 (3.1)	48 (12.3)	170 (43.7)	157 (40.4)	1.80	0.811
Have bread/noodles made with wheat as a meal	21 (5.4)	69 (17.6)	168 (43)	111 (28.4)	22 (5.6)	2.89	0.943
Have dairy products such as milk, yogurt, cheese	15 (3.8)	32 (8.1)	113 (28.8)	170 (43.3)	63 (16)	2.40	0.978
Eat fast food	5 (1.3)	26 (6.6)	117 (29.8)	203 (51.7)	42 (10.7)	2.36	0.809
Have snacks such as cakes, candies, soft drinks	19 (4.9)	50 (12.8)	124 (31.7)	158 (40.4)	40 (10.2)	2.62	0.995
Have snacks such as fruit, nuts	10 (2.6)	29 (7.4)	73 (18.6)	161 (41.1)	119 (30.4)	2.11	1.003
Eat out	37 (9.5)	92 (23.5)	132 (33.8)	107 (27.4)	23 (5.9)	3.03	1.060
Have ready-prepared meals (HMRs)	11 (2.8)	51 (13.1)	106 (27.2)	161 (41.4)	60 (15.4)	2.47	0.996
Skip breakfast	130 (33.4)	65 (16.7)	64 (16.5)	79 (20.3)	51 (13.1)	3.37	1.449
Skip lunch	8 (2.1)	23 (5.9)	67 (17.2)	101 (25.9)	191 (49)	1.86	1.032
Skip dinner	5 (1.3)	12 (3.1)	43 (10.9)	116 (29.5)	217 (55.2)	1.66	0.884
Overeat	6 (1.5)	26 (6.6)	115 (29.3)	194 (49.4)	52 (13.2)	2.34	0.845

**Table 3:** Results of the comparisons of the students’ physical activities, alcoholic beverage consumption, and dietary habits based on sex, academic year, and residence type.

I Usually…per a Week	Male		Female			Academic Year	Residence Type
	Mean	SD Mean		SD	t-Value	F-Value	F-Value
Exercise regularly	2.21	1.018	1.67	0.766	5.823 ***	2.019	0.248
Exercise more than 30 min	2.14	1.040	1.60	0.780	5.701 ***	4.245 **	0.174
Drink alcoholic beverages	1.82	0.798	1.75	0.783	0.902	4.074 **	1.514
Take dietary supplements	2.06	1.417	1.94	1.295	0.815	4.343 **	0.749
Eat when I am stressed	2.09	0.915	2.30	0.879	−2.263 *	0.745	0.767
Eat more than two pieces of fresh fruit	1.94	0.984	2.16	1.022	−2.168 *	0.619	11.293 ***
Eat meat	3.35	0.998	2.77	0.863	6.173 ***	0.458	0.264
Eat fish and seafood	1.94	0.828	1.67	0.777	3.311 **	1.886	8.437 ***
Have bread/noodles made with wheat as a meal	2.85	0.943	2.93	0.942	−0.917	0.645	1.109
Have dairy products such as milk, yogurt, cheese	2.44	0.917	2.38	1.033	0.531	0.353	2.432
Eat fast food	2.44	0.784	2.30	0.819	1.809	248	1.720
Have snacks such as cakes, candies, soft drinks	2.49	1.005	2.73	0.984	−2.368 *	0.679	0.668
Have snacks such as fruit, milk, nuts	2.04	0.979	2.16	1.026	−1.218	3.999 **	7.936 ***
Eat out	3.03	1.068	3.06	1.043	−0.209	0.478	2.450
Have ready-prepared meals (HMRs)	2.44	0.982	2.49	1.011	−0.494	0.252	2.360
Skip breakfast	3.30	1.415	3.44	1.486	−0.977	0.174	7.894 ***
Skip lunch	1.75	0.904	1.95	1.121	−1.917	0.649	4.408 **
Skip dinner	1.55	0.820	1.73	0.917	−2.068 *	0.922	2.088
Overeat	2.38	0.860	2.30	0.838	0.926	2.078	0.919

* *p* < 0.05, ** *p* < 0.01, *** *p* < 0.001.

**Table 4:** Measurement items for the Perceived Stress Scale (PSS-10).

In the Last Month, How Often Have You	Mean	SD	Helplessness	Self-Efficacy
Q1 felt anxious about something that happened unexpectedly?	1.85	0.836	0.752	
Q2 felt unable to control the important things in your life?	1.31	0.959	0.845	
Q3 felt nervous and stressed?	1.79	1.082	0.808	
Q6 found that you could not cope with all the things that you had to do?	1.76	0.958	0.657	
Q9 been angered because of things that were outside of your control?	1.60	1.048	0.752	
Q10 felt difficulties were piling up so high that you could not overcome them?	1.60	1.062	0.818	
Q4 felt confident about your ability to handle your personal problems?	2.09	0.947		0.795
Q5 felt that things were going your way?	2.17	0.850		0.838
Q7 been able to control irritations in your life?	1.70	0.946		0.561
Q8 felt that you were on top of things?	2.56	0.936		0.733
Eigen value			3.694	2.259
Cronbach’s alpha			0.866	0.711

**Table 5:** Results of the comparisons of physical activities, alcoholic beverage consumption, and dietary habits of low- and high-stress groups.

	Low Stress		High Stress		t-Value
I Usually…per a Week	Mean SD				
Exercise regularly	2.02	0.981	1.81	0.854	2.137 *
Exercise more than 30 min	1.93	1.008	1.76	0.854	1.741
Drink alcoholic beverages	1.75	0.777	1.81	0.785	−0.715
Take dietary supplements	1.85	1.307	2.11	1.362	−1.878
Eat when I am stressed	1.94	0.781	2.43	0.933	−5.556 ***
Eat more than two pieces of fresh fruit	2.07	1.036	2.03	0.975	0.344
Eat meat	3.12	0.982	2.93	0.956	2.002 *
Eat fish and seafood	1.81	0.767	1.76	0.822	0.560
Have bread/noodles made with wheat as a meal	2.77	0.896	3.01	0.964	−2.445 *
Have dairy products such as milk, yogurt, cheese	2.44	1.020	2.37	0.895	0.746
Eat fast food	2.27	0.752	2.44	0.854	−2.026 *
Have snacks such as cakes, candies, soft drinks	2.42	0.895	2.81	1.048	−3.909 ***
Have snacks such as fruit, milk, nuts	2.09	0.983	2.11	0.989	−0.187
Eat out	2.95	1.092	3.13	1.028	−1.653
Have ready-prepared meals (HMRs)	2.22	0.908	2.72	1.039	−4.897 ***
Skip breakfast	3.31	1.512	3.40	1.395	−0.643
Skip lunch	1.68	0.984	2.03	1.052	−3.328 **
Skip dinner	1.51	0.804	1.81	0.938	−3.422 **
Overeat	2.21	0.871	2.45	0.809	−2.779 **

* *p* < 0.05, ** *p* < 0.01, *** *p* < 0.001.

## References

[1] Hedley A.A., Ogden C.L., Johnson C.L., Carroll M.D., Curtin L.R., Flegal K.M., Hedley A.A., Ogden C.L., Johnson C.L., Carroll M.D. (2004). Prevalence of overweight and obesity among US children, adolescents, and adults, 1999–2002. JAMA J. Am. Med. Assoc..

[2] American College Health Association (2009). American College Health Association-National College Health Assessment Spring 2008 Reference Group Data Report (Abridged). J. Am. Coll. Health.

[3] Sa J., Choe S., Cho B.-Y., Chaput J.-P., Kim G., Park C.-H., Chung J., Choi Y., Nelson B., Kim Y. (2020). Relationship between sleep and obesity among U.S. and South Korean college students. BMC Public Health.

[4] Crombie A.P., Ilich J.Z., Dutton G.R., Panton L.B., Abood D.A. (2009). The freshman weight gain phenomenon revisited. Nutr. Rev..

[5] Fedewa M.V., Das B.M., Evans E.M., Dishman R.K. (2014). Change in weight and adiposity in college students: A systematic review and meta-analysis. Am. J. Prev. Med..

[6] Wengreen H.J., Moncur C. (2009). Change in diet, physical activity, and body weight among young-adults during the transition from high school to college. Nutr. J..

[7] Deshpande S., Basil M.D., Basil D.Z. (2009). Factors Influencing Healthy Eating Habits Among College Students: An Application of the Health Belief Model. Health Mark. Q..

[8] Barnes S.P., Brown K.M., McDermott R.J., Bryant C.A., Kromrey J. (2012). Perceived Parenting Style and the Eating Practices of College Freshmen. Am. J. Health Educ..

[9] Butler S.M., Black D.R., Blue C.L., Gretebeck R.J. (2004). Change in Diet, Physical Activity, and Body Weight in Female College Freshman. Am. J. Health Behav..

[10] Graham M.A., Jones A.L. (2002). Freshman 15: Valid theory or harmful myth?. J. Am. Coll. Health.

[11] Abraham S., Noriega B.R., Shin J.Y. (2018). College Students Eating Habits and Knowledge of Nutritional Requirements. J. Nutr. Hum. Health.

[12] Morse K.L., Driskell J.A. (2009). Observed sex differences in fast-food consumption and nutrition self-assessments and beliefs of college students. Nutr. Res..

[13] Marquis M. (2005). Exploring convenience orientation as a food motivation for college students living in residence halls. Int. J. Consum. Stud..

[14] Driskell J.A., Kim Y.-N., Goebel K.J. (2005). Few Differences Found in the Typical Eating and Physical Activity Habits of Lower-Level and Upper-Level University Students. J. Am. Diet. Assoc..

[15] Driskell J.A., Meckna B.R., Scales N.E. (2006). Differences exist in the eating habits of university men and women at fast-food restaurants. Nutr. Res..

[16] Nicklas T.A., Baranowski T., Cullen K.W., Berenson G. (2001). Eating Patterns, Dietary Quality and Obesity. J. Am. Coll. Nutr..

[17] Dinger M.K., Waigandt A., Dinger M.K., Waigandt A. (1997). Dietary intake and physical activity behaviors of male and female college students. Am. J. Health Promot..

[18] Tam R., Yassa B., Parker H., O’Connor H., Allman-Farinelli M. (2017). University students’ on-campus food purchasing behaviors, preferences, and opinions on food availability. Nutrition.

[19] Racette S.B., Deusinger S.S., Strube M.J., Highstein G.R., Deusinger R.H. (2005). Weight changes, exercise, and dietary patterns during freshman and sophomore years of college. J. Am. Coll. Health.

[20] Ross S.E., Niebling B.C., Heckert T.M. (1999). Sources of stress among college students. Coll. Stud. J..

[21] Zellner D.A., Loaiza S., Gonzalez Z., Pita J., Morales J., Pecora D., Wolf A. (2006). Food selection changes under stress. Physiol. Behav..

[22] Wansink B., Cheney M.M., Chan N. (2003). Exploring comfort food preferences across age and gender. Physiol. Behav..

[23] O’Connor D.B., Jones F., Conner M., McMillan B., Ferguson E. (2008). Effects of daily hassles and eating style on eating behavior. Health Psychol..

[24] National Research Council (2008). Recognition and Alleivation of Distress in Laboratory Animals.

[25] Papier K., Ahmed F., Lee P., Wiseman J. (2015). Stress and dietary behaviour among first-year university students in Australia: Sex differences. Nutrition.

[26] Wallis D.J., Hetherington M.M. (2009). Emotions and eating. Self-reported and experimentally induced changes in food intake under stress. Appetite.

[27] Maxwell Annette E., El Ansari W., Mikolajczyk Rafael T. (2009). Food consumption frequency and perceived stress and depressive symptoms among students in three European countries. Nutr. J..

[28] Willenbring M.L., Levine A.S., Morley J.E. (1986). Stress Induced Eating and Food Preference in Humans: A Pilot Study. Int. J. Eat. Disord..

[29] Oliver G., Wardle J. (1999). Perceived Effects of Stress on Food Choice. Physiol. Behav..

[30] Roman G., Rusu A., Graur M., Creteanu G., Morosanu M., Radulian G., Amorin P., Timar R., Pircalaboiu L., Bala C. (2019). Dieatary patterns and their association with obesity: A cross-sectional study. Acta Endocrinol. (1841-0987).

[31] Cespedes E.M., Hu F.B. (2015). Dietary patterns: From nutritional epidemiologic analysis to national guidelines. Am. J. Clin. Nutr..

[32] Acampado E., Valenzuela M. (2018). Physical activity and dietary habits of Filipino college students. Kinesiology.

[33] Alzamil H.A., Alhakbany M.A., Alfadda N.A., Almusallam S.M., Al-Hazzaa H.M. (2019). A Profile of Physical Activity, Sedentary Behaviors, Sleep, and Dietary Habits of Saudi College Female Students. J. Fam. Community Med..

[34] Sheldon C., Tom K., Robin M. (1983). A Global Measure of Perceived Stress. J. Health Soc. Behav..

[35] Maroufizadeh S., Foroudifard F., Navid B., Ezabadi Z., Sobati B., Omani-Samani R. (2018). The Perceived Stress Scale (PSS-10) in women experiencing infertility: A reliability and validity study. Middle East Fertil. Soc. J..

[36] Cohen S. (1988). Perceived stress in a probability sample of the United States. The Social Psychology of Health.

[37] Lee E.-H. (2012). Review of the Psychometric Evidence of the Perceived Stress Scale. Asian Nurs. Res..

[38] Nielsen T., Dammeyer J. (2019). Measuring higher education students’ perceived stress: An IRT-based construct validity study of the PSS-10. Stud. Educ. Eval..

[39] IBM Corporation (2017). IBM Statistical Package for the Social Sciences for Windows, version 25.0.

[40] Lim J.U., Lee J.H., Kim J.S., Hwang Y.I., Kim T.H., Lim S.Y., Yoo K.H., Jung K.S., Kim Y.K., Rhee C.K. (2017). Comparison of World Health Organization and Asia-Pacific body mass index classifications in COPD patients. Int. J. Chron. Obstruct. Pulmon. Dis..

[41] Tajik E., Latiffah A.L., Awang H., Siti Nur’Asyura A., Chin Y.S., Azrin Shah A.B., Patricia Koh C.H., Mohd Izudin Hariz C.G. (2016). Unhealthy diet practice and symptoms of stress and depression among adolescents in Pasir Gudang, Malaysia. Obes. Res. Clin. Pract..

[42] Weinstein S.E., Shide D.J., Rolls B.J. (1997). Changes in Food Intake in Response to Stress in Men and Women: Psychological Factors. Appetite.

[43] Mahoney C.R., Giles G.E., Marriott B.P., Judelson D.A., Glickman E.L., Geiselman P.J., Lieberman H.R. (2019). Intake of caffeine from all sources and reasons for use by college students. Clin. Nutr..

[44] Brevard P.B., Ricketts C.D. (1996). Residence of college students affects dietary intake, physical activity, and serum lipid levels. J. Am. Diet. Assoc..

[45] Emond M., Ten Eycke K., Kosmerly S., Robinson A.L., Stillar A., Van Blyderveen S. (2016). The effect of academic stress and attachment stress on stress-eaters and stress-undereaters. Appetite.

[46] Lee G., Han K., Kim H. (2017). Risk of mental health problems in adolescents skipping meals: The Korean National Health and Nutrition Examination Survey 2010 to 2012. Nurs. Outlook.

[47] Carels R.A., Young K.M., Coit C., Clayton A.M., Spencer A., Wagner M. (2008). Skipping meals and alcohol consumption: The regulation of energy intake and expenditure among weight loss participants. Appetite.

[48] Bentley C., Gratwick-Sarll K., Harrison C., Mond J. (2015). Sex differences in psychosocial impairment associated with eating disorder features in adolescents: A school-based study. Int. J. Eat. Disord..

[49] Scott G., Phillipa H., Deborah M., Jonathan M.M., Siân A.M., Bryan R., Robin M., Susan J.P. (2016). Sex differences in the relationships between body dissatisfaction, quality of life and psychological distress. Aust. N. Z. J. Public Health.

